# Lower limb kinematic, kinetic and spatial-temporal gait data for healthy adults using a self-paced treadmill

**DOI:** 10.1016/j.dib.2020.106613

**Published:** 2020-12-09

**Authors:** Shayan Bahadori, Jonathan Mark Williams, Thomas W Wainwright

**Affiliations:** a Orthopaedic Research Institute, Bournemouth University, Bournemouth, Dorset, United Kingdom; b Faculty of Health and Social Sciences, Bournemouth University, Bournemouth, Dorset, United Kingdom; c Physiotherapy Department, University Hospitals Dorset NHS Foundation Trust, Bournemouth, Dorset, United Kingdom

**Keywords:** Gait, Kinematic, Kinetic, Spatial-temporal

## Abstract

Through gait analysis, gait phases can be identified, the kinematic and kinetic parameters of human gait events can be determined, and quantitative evaluation can be undertaken. This data article is the first to report a comprehensive data set on a large cohort of healthy participants. Individual strides were determined from vertical force data and all kinematics and kinetic data separated into strides. Local minima and maxima were determined respectively for each anatomical region and the mean calculated for twenty of the 25 strides. When twenty strides were not available the mean of ten strides was used. Stride data were time normalised so one stride was represented by 100%. In addition to the local maxima and minima, the kinematic- and kinetic-time curves were explored and used to determine the mean kinematic-time and kinetic-time curves across all trials and participants (∼1800 gait cycles) to provide mean±sd kinematic- and kinetic-time curves for each of the anatomical regions.

## Specifications Table

**Table:** 

Subject	Biomedical engineering
Specific subject area	Human gait data for healthy adults
Type of data	Table Graph Figure
How data were acquired	Make and model and of the instruments used: Gait Real-time Analysis Interactive Laboratory (GRAIL) system – Motekforce Link
Data format	Raw Analysed
Parameters for data collection	The Human Body Model (HBM) lower body marker set [1] was used and participants were asked to walk for minimum of five minutes. Marker data were low-pass filtered with a second order Butterworth filter with a cut-off frequency of 6 Hz. Gait event detection was calculated based on foot markers [2].
Description of data collection	Participants walked on a self-paced treadmill, wore comfortable shoes and tight clothing, and were fitted with 25 passive markers using HBM. Knee and ankle width was measured using a joint ruler for the HBM model. Participants were given a minimum of five minutes to adapt to the self-paced treadmill walking. Following the acclimatisation, participants were asked to walk for a minimum of five minutes, and three sets of 25 gait cycles were recorded with the participant blinded to the timing of the recordings.
Data source location	Institution: Bournemouth University Orthopaedic Research Institute City/Town/Region: Bournemouth Country: United Kingdom
Data accessibility	In a public repository Repository name: Mendeley Data Direct URL to data: http://dx.doi.org/10.17632/wwnvw28n2m.1

## Value of the Data

• Human gait requires intact cognition and executive control, and depends on inputs from the nervous, musculoskeletal, and cardiorespiratory systems. Gait disorders may lead to disability, falls and injuries, and result in a marked reduction in the quality of life. Gait analysis is therefore an essential part of the diagnosis of various medical disorders, and the assessment of patient progress during rehabilitation and recovery.
• Gait analysis facilitates are expensive and therefore are frequently unavailable to many researchers, students, and professionals who use biomechanical data for their work. Such individuals may work across areas such as medicine, rehabilitation, sports and exercise science, public health, engineering, and technology. Providing open source data on normal participants can therefore be hugely valuable.
• The data presented in this study, acquired from 100 “normal” participants of different ages, will provide a valuable dataset for future work involving locomotion in humans. Specific applications may include its use as a normative reference when analysing abnormal or pathological gait, or to develop algorithms for animated models, new prosthetics, bipedal robotic devices, and active exoskeletons.
  By providing such a comprehensive dataset to an international audience, we hope that this will allow colleagues without access to a gait laboratory to further their academic and clinical projects.

## 1 Data Description

One-hundred healthy adults participated in the study, (aged from 21 to 79 years, mean±sd; 41.9±15.3, body mass 71.4±15.1 kg, height 172.5±9.5 cm; 51 males, 49 females). Participant's characteristics are summarized in **Table 1.**

Results for all participants including the mean±sd of the spatial-temporal, kinematics and kinetic gait parameters are given in **Tables 2, 3, 4**. Results for the local maxima and minima of the kinematic- and kinetic-time curves for all participants are given in **Tables 5 – 14.**

Recent publications have assessed the capability [3], [4], [5] and protocol [6] of the GRAIL system in gait analysis as well as its day to day reliability [7,8].

Table 2 - Joint kinematic gait data for the 100 participants. Kinematic data are in degrees (°) and evaluates the joints’ range of motion, time, and distance parameter without consideration of the forces involved.

Table 3 - Joint kinetic gait data for the 100 participants. Kinetic data are in Newton meter per kilogram (Nm/kg). This assessment quantifies the forces and muscle activity that occurs during gait.

Table 4 - Spatial temporal gait data for the 100 participants. Data includes average walking speed in meters per second (m/s), average step length for right and left side in meters (m), average stride time for right and left side in seconds (s), average stance time for right and left side in seconds (s), and average swing time for right and left side in seconds (s). Average walking speed was derived by time and distance required for the participant to complete 25 cycles [9]. Step length is the distance between heel strike of one foot and the other foot [9]. Stride time is the duration of one gait cycle, defined as the time between heel strikes on one foot [9]. Stance time is the amount of time that passes during the stance phase of one extremity in a gait cycle [9]. It includes single support and double support. Swing time is the amount of time that passes during the swing phase of one extremity in a gait cycle [9].

Table 5 - Left ankle flexion-extension movement-time curve for the 100 participants. Data are in degrees (°). Stride for each participants data were time normalised so one stride was represented by 100% (one complete cycle). Initial point (0%) up to 60% represent the stance phase and from 60% to 100% represents the swing phase for each participant.

Table 6 - Left hip abduction-adduction movement-time curve for the 100 participants. Data are in degrees (°). Stride for each participants data were time normalised so one stride was represented by 100% (one complete cycle). Initial point (0%) up to 60% represent the stance phase and from 60% to 100% represents the swing phase for each participant.

Table 7 - Left hip flexion-extension movement-time curve for the 100 participants. Data are in degrees (°). Stride for each participants data were time normalised so one stride was represented by 100% (one complete cycle). Initial point (0%) up to 60% represent the stance phase and from 60% to 100% represents the swing phase for each participant.

Table 8 - Left hip rotation movement-time curve for the 100 participants. Data are in degrees (°). Stride for each participants data were time normalised so one stride was represented by 100% (one complete cycle). Initial point (0%) up to 60% represent the stance phase and from 60% to 100% represents the swing phase for each participant.

Table 9 - Left knee flexion-extension movement for the 100 participants. Data are in degrees (°). Stride for each participants data were time normalised so one stride was represented by 100% (one complete cycle). Initial point (0%) up to 60% represent the stance phase and from 60% to 100% represents the swing phase for each participant.

Table 10 - Right ankle flexion-extension movement-time curve for the 100 participants. Data are in degrees (°). Stride for each participants data were time normalised so one stride was represented by 100% (one complete cycle). Initial point (0%) up to 60% represent the stance phase and from 60% to 100% represents the swing phase for each participant.

Table 11 - Right hip abduction-adduction movement-time curve for the 100 participants. Data are in degrees (°). Stride for each participants data were time normalised so one stride was represented by 100% (one complete cycle). Initial point (0%) up to 60% represent the stance phase and from 60% to 100% represents the swing phase for each participant.

Table 12 - Right hip flexion-extension movement-time curve for the 100 participants. Data are in degrees (°). Stride for each participants data were time normalised so one stride was represented by 100% (one complete cycle). Initial point (0%) up to 60% represent the stance phase and from 60% to 100% represents the swing phase for each participant.

Table 13 - Right hip rotation movement-time curve for the 100 participants. Data are in degrees (°). Stride for each participants data were time normalised so one stride was represented by 100% (one complete cycle). Initial point (0%) up to 60% represent the stance phase and from 60% to 100% represents the swing phase for each participant.

Table 14 - Right knee Flexion-extension movement-time curve for the 100 participants. Data are in degrees (°). Stride for each participants data were time normalised so one stride was represented by 100% (one complete cycle). Initial point (0%) up to 60% represent the stance phase and from 60% to 100% represents the swing phase for each participant.

### 1.1 Code availability

Kinematic, kinetic and spatial-temporal gait parameters for all participants are exported as a .CSV file and analysed in Matlab R2017a (the Mathworks Inc., USA) **(Supplementary File 1).**

## 2 Experimental Design, Materials and Methods

### 2.1 Instrumentation and protocol

Participants walked on a split-belt instrumented treadmill, with 160 semi-cylindrical projection screen of which the optical flow was continuously matched to the walking speed (GRAIL, Motek Medical BV, the Netherlands). Force sensors underneath each belt (50 × 200 cm) recorded the ground reaction forces and moments. 3D locations of the lower body were tracked using passive markers and a 10-camera Vicon MX optical infrared system (Oxford Metrics, UK), synced at 200 Hz to the force data. Lower body joint kinematics and kinetics were calculated in real-time using the Human Body Model (HBM; Motek Medical BV).

**Fig. 1:**
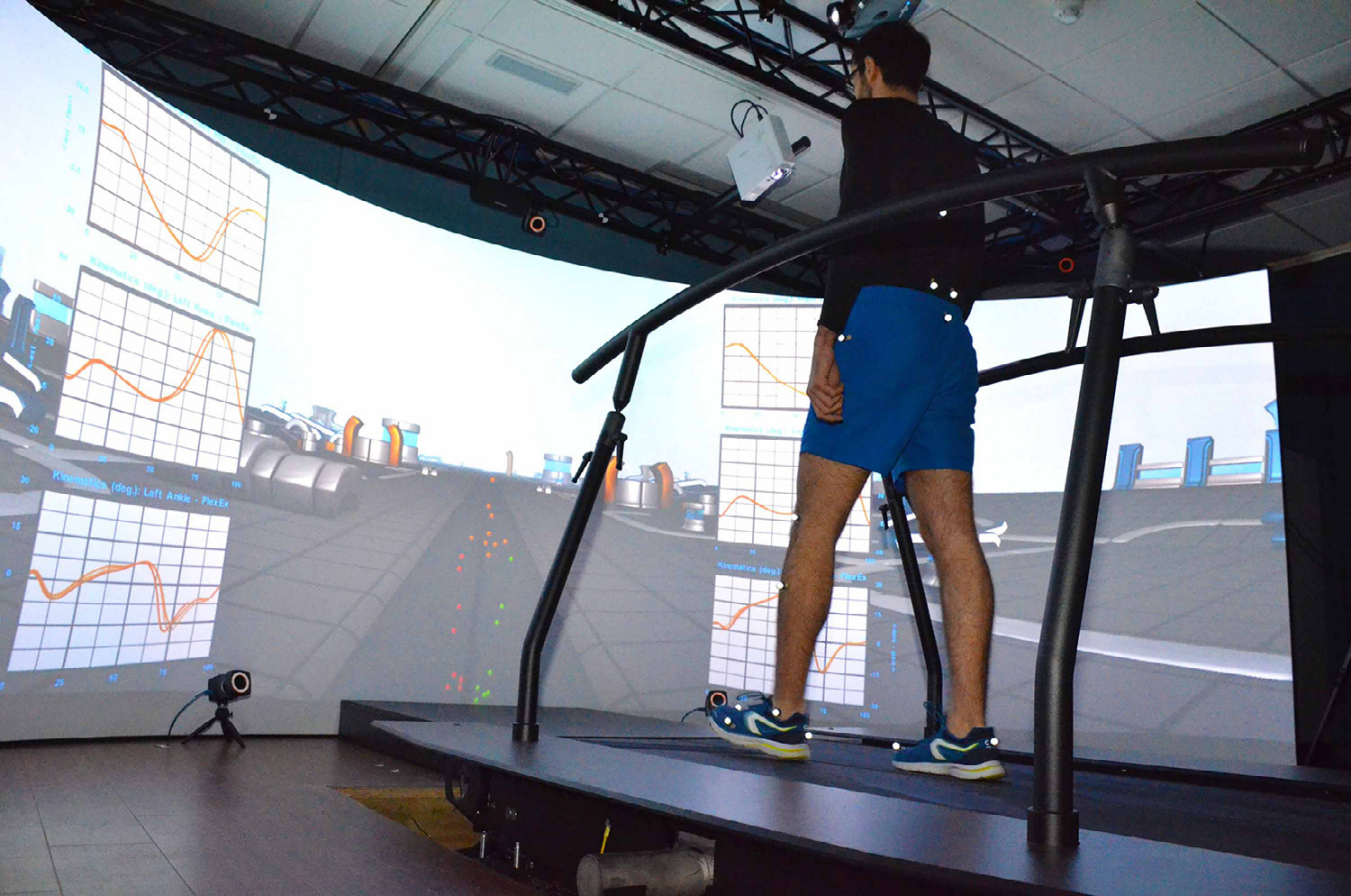
Orthopaedic Research Institute gait laboratory. Schematic illustrates the locations for VICON cameras, VR screen, projectors, and self-paced treadmill.

**Fig. 2:**
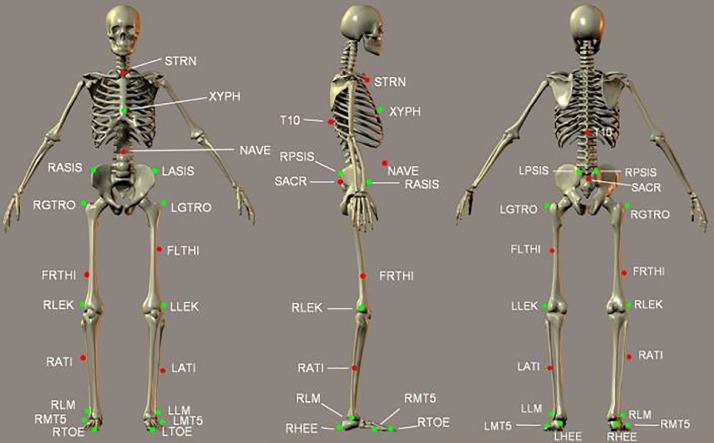
Diagram of markers.

Participants wore comfortable shoes and tight clothing (such as cycling shorts or leggings) (Fig. 1). They were fitted with 25 passive reflective markers using the Human Body Model (HBM) lower body marker set [1] as detailed in **Table 15 and** Fig. 2. Knee and ankle widths were measured using a joint ruler for the HBM model. Participants wore the safety harness and were given a minimum of five minutes to adapt to the self-paced treadmill walking [4, 5]. Following the acclimatisation, participants were asked to walk for a minimum of five minutes and three sets of 25 gait cycles [5, 10] were recorded with participants blinded to the timing of the recordings. Treadmill and recordings of gait cycles were controlled using D-Flow software (version 3.26, Motekforce Link, Amsterdam, the Netherlands) [11].

**Table 15:** Markers used in the Human Body Model.

## 3 Steps of Data Recording Procedure

### 3.1 Participant preparation

Participants are required to wear very tight non-reflective clothing onto which 25 reflective markers are placed using double sided tape. Fig. 2 shows the exact placements of all markers in the HBM lower body model. Special attention should be payed to the placement of the markers printed in green (bold in Table 15); these are used during initialization to define the biomechanical skeleton.

### 3.2 Capture and processing a range of motion trial

Prior to actual data capture participants performed a series of participant calibration steps, known as a range of motion trail (ROM). This ROM trial is recorded and processed in Vicon Nexus in order to minimize the real time labelling errors of the HBM. Capturing and processing a ROM trial using Nexus, steps are as follow:

1. Start a new session in the database and make sure it is active (highlighted).
2. Ensure Nexus runs in ‘Live’ mode.
3. Using the subject tab, create a new participant from a ‘Labelling Skeleton’ button.
4. Browse to the ‘LowerLimb HBM_N2.vst’ file and then enter the name of the participant. The new participant appears in the Subjects pane.
5. Go to the Tools pane and open the ‘Subject Preparation’ tab.
6. ‘Zero level’ the force plates via the ‘Hardware’ tab (make sure no weight is exerted on the force plates).
7. Prepare the participant for the ROM trial by having them ready in the middle of the treadmill.
8. Put on the safety harness, making sure markers are not blocked by the harness.
9. Instruct the participant to do the following once everything is set up to record:
  a. You'll start in a T-pose: keep your hands at shoulder height, palms facing down and feet at hip width distance (at either side of the treadmill gap), facing forward.
  b. The treadmill will be activated and speed will increase until your comfortable speed is reached.
  c. You'll walk for a few seconds.
  d. After a few seconds the treadmill will be stopped.
10. When the participant is standing in T-pose hit ‘Start’ in Nexus to start capturing the ROM.
11. Turn on the treadmill in D-Flow.
12. Let the participant walk for a few seconds at his/her comfortable walking speed.
13. Stop the recording in Nexus (Nexus will switch to Offline mode and the recorded trial will automatically be opened).
14. Stop the treadmill in D-Flow.
15. Processing the ROM trial starts by going to the ‘Pipeline’ tab in the ‘Tools’ pane and selecting the ‘ Calibrate Autolabel from ROM’ pipeline under ‘Current Pipeline’.
16. Run the pipeline steps. It is recommended to run each step of the pipeline separately instead of running the entire pipeline at once. This way it will be possible to check the data every time a part of the process is carried out. Right click on the specific operation and choose ‘Run selected Op’.
  a. Reconstruct: the reconstructed markers will appear in the 3D perspective view
  b. Autolabel Static Frame (Autolabel Static): compare marker labelling with the picture. (If markers are incorrectly labelled, click on the ‘Label/Edit’ button, and label the markers manually by first selecting a label from the list and then selecting the correct marker in the 3D perspective view).

**Fig. 3:**
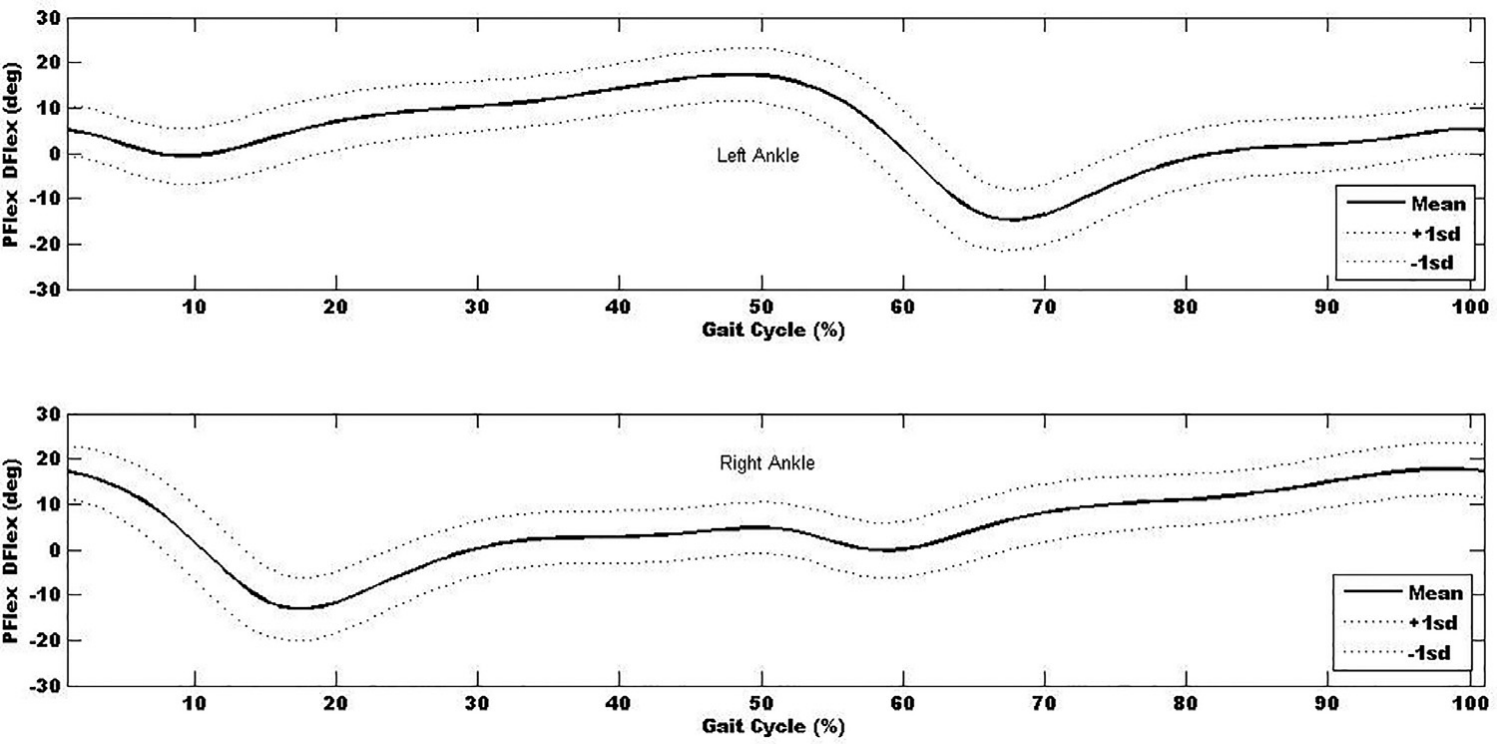

**Fig. 4:**
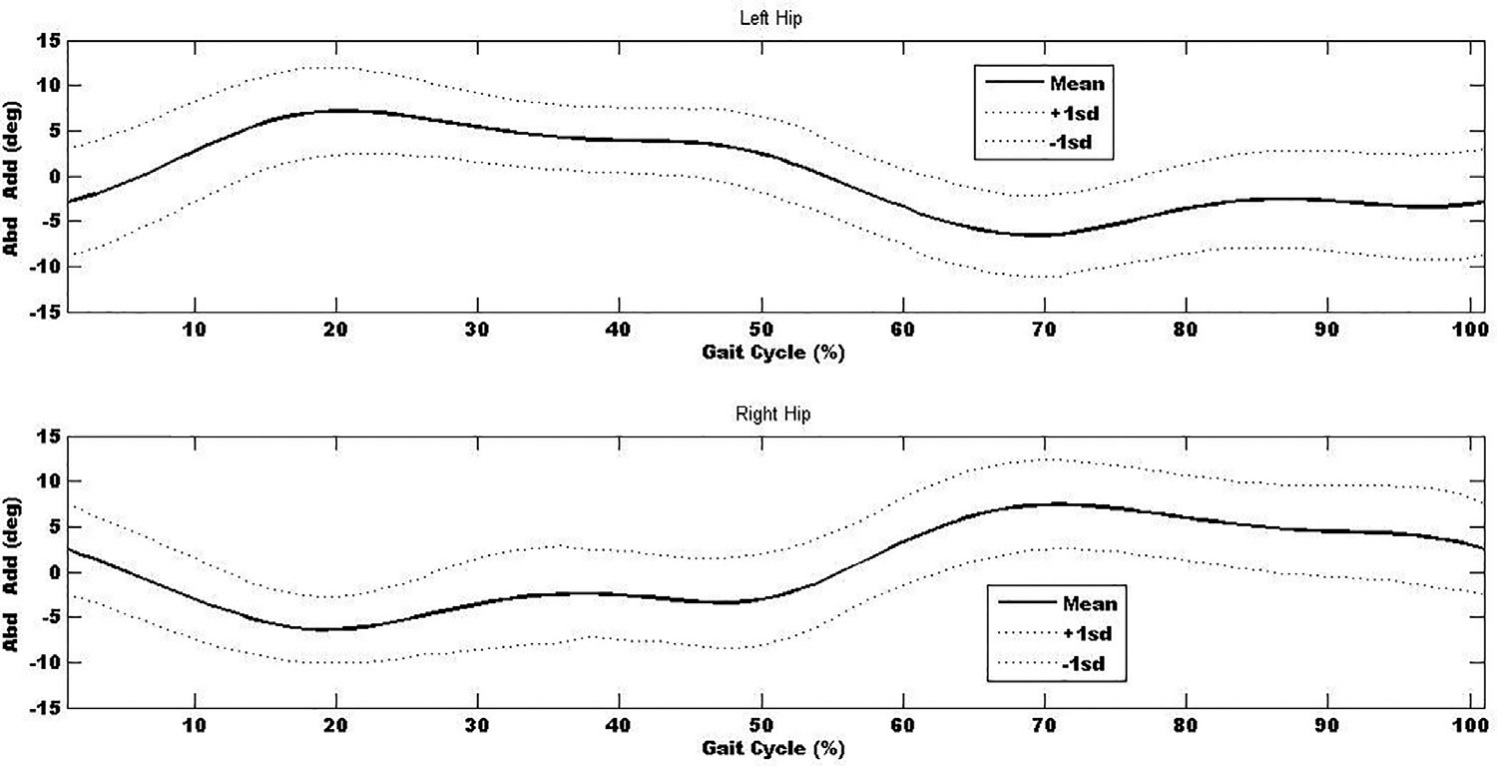

**Fig. 5:**
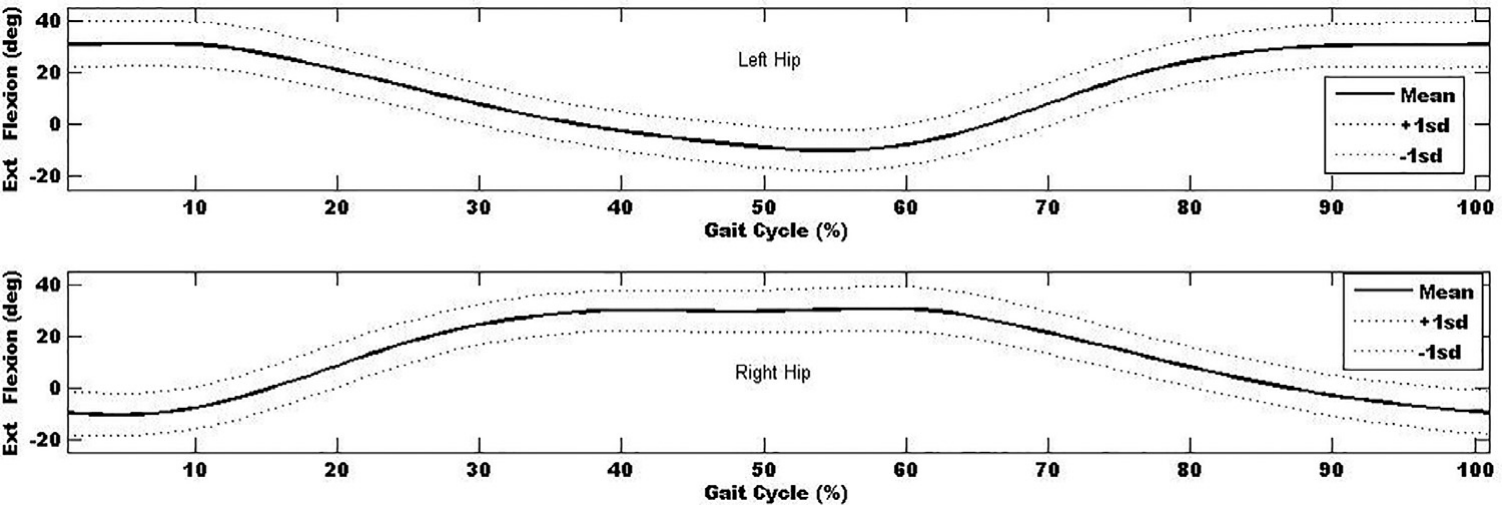

**Fig. 6:**
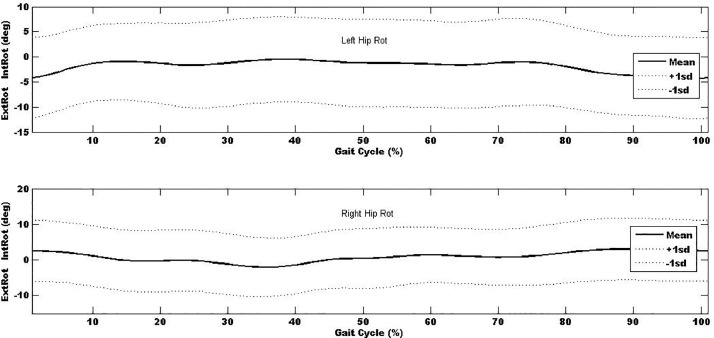

**Fig. 7:**
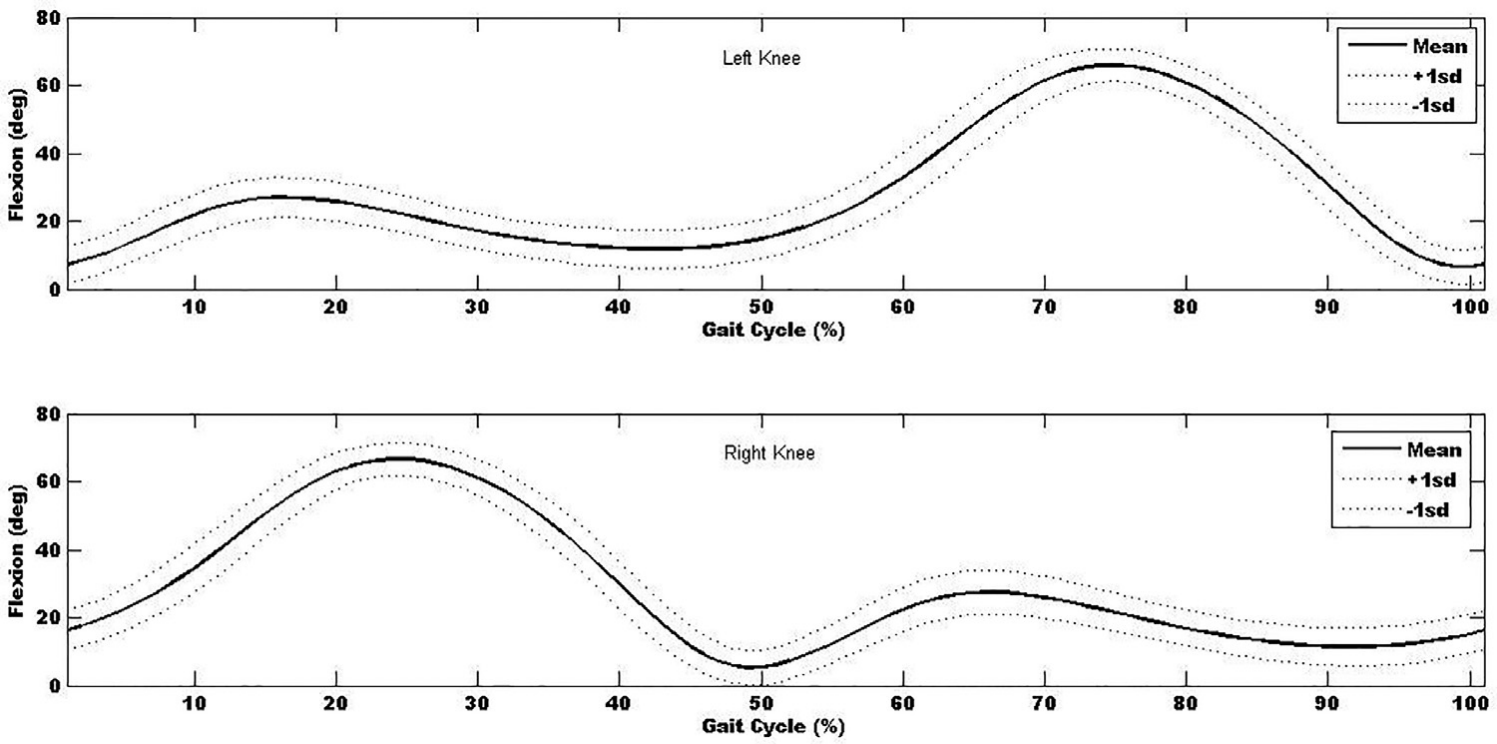
  c. Scale subject (Scale Subject VSK): Scales the labelling skeleton to be the same size as a labelled set of reconstructions on a particular frame. Therefore a client specific scaling of the segments of the calibration file is created.
  d. Functional Subject Calibration: This operation will calibrate the subject's bone lengths, joint locations and marker locations using the whole trial.
17. After running the pipeline, make sure that Nexus is set to ‘Live’ mode and provides visibly stable marker data.
  a. If the marker data is not stable go to the ‘System’ pane on the left hand side and select ‘Vicon System’. Check if the settings: Processing level under Core processor is set to ‘Label’. Minimum Cameras to Start trajectory is set to 2. Minimum Cameras to Continue trajectory is set to

### 3.3 Recording gait data in gait applications with D-Flow

1. In the menu tab, go to File, Open Applications from MM Gait version, then select ‘Gait Feedback Module’.
2. Go to the ‘Subject info’ tab in the Runtime Console and enter the Gender and ankle and knee width of the participant.
3. Have the participant stand in a T-pose in the centre of the treadmill: Press the ‘Calibrate HBM’ button.
4. Give participant an ID in the runtime console. The data will then be automatically saved in a folder with the same name as the ID.
5. Start the application and have the participant walk at a comfortable speed for at least 5 minutes before start recording data.
6. Start data recording by hitting the ‘Start recording gait data’ button.
7. Stop the recording after acquiring the desired amount of data. It is recommended to collect three sets of 25 cycles.

### 3.4 Exporting gait data using GOAT

1. Open the GOAT software.
  a. It is possible to add information such as name, gender, date and diagnosis here. As per our ethics guidelines, all of the data collected for this study were non-identifiable
2. Go to ‘File’, and then select ‘Export’ to save as ‘CSV’.

## 4 Data Processing

Marker data were low-pass filtered with a second order Butterworth filter with a cut-off frequency of 6 Hz. Gait events detection were calculated based on foot markers [2]. Walking speed was exported from the GRAIL treadmill output [3] using the GOAT. Kinematic, kinetic and spatial-temporal gait parameters were exported to a .CSV file and analysed in Matlab R2017a. All raw data were transferred to Matlab for processing using a bespoke algorithm and all kinematics and kinetic data separated into strides. Local minima and maxima were determined respectively for each anatomical region and means calculated for twenty of the 25 strides. When twenty strides were not available the mean of ten strides were used. Stride data were time normalised so one stride was represented by 100%.

In additional to the local maxima and minima, the kinematic- and kinetic-time curves were explored and used to determine the mean kinematic-time and kinetic-time curves across all trials and participants (∼1800 gait cycles) to provide mean±sd kinematic- and kinetic-time curves for each of the anatomical regions. Mean±sd kinematic-time graph for a single stride for each anatomical region are provided in Figs. 3, 4, 5, 6, 7, which indicates that the data points are close to the mean values.

## Ethics Statement

Each participant was provided with information about the study and asked to provide written informed consent. The study was approved by Bournemouth University Ethical Committee (Ref: 15005).

## Declaration of Competing Interest

The authors declare that they have no known competing financial interests or personal relationships which have, or could be perceived to have, influenced the work reported in this article.

## Appendix Supplementary materials

Image, application 1

Image, application 2
