# Supplementary material for: Lower limb kinematic, kinetic and spatial-temporal gait data for healthy adults using a self-paced treadmill
Source: Data Brief. 2020 Dec 9;34:106613. doi: 10.1016/j.dib.2020.106613 (PMC7773874; doi:10.1016/j.dib.2020.106613)
Supplement: Supplementary file 2 [file mmc2.docx]

Lsteplength = data(:,40);

ix = (Lsteplength==0); Lsteplength(ix) = [];

Lstridetime = data(:,43);

ix = (Lstridetime==0); Lstridetime(ix) = [];

Lstancetime = data(:,39);

ix = (Lstancetime==0); Lstancetime(ix) = [];

Lswingtime = data(:,44);

ix = (Lswingtime==0); Lswingtime(ix) = [];

Rsteplength = data(:,251);

ix = (Rsteplength==0); Rsteplength(ix) = [];

Rstridetime = data(:,254);

ix = (Rstridetime==0); Rstridetime(ix) = [];

Rstancetime = data(:,250);

ix = (Rstancetime==0); Rstancetime(ix) = [];

Rswingtime = data(:,255);

ix = (Rswingtime==0); Rswingtime(ix) = [];

walksp = data(:,285);

RotPelvicObl = data(:,268);

RotPelvicRot = data(:,269);

RotPelvicTil = data(:,270);

LHipFlex = data(:,263);

LHipAbAd = data(:,262);

LHipRot = data(:,264);

RHipFlex = data(:,276);

RHipAbAd = data(:,275);

RHipRot = data(:,277);

LKneeFlex = data(:,266);

LKneeAbAd = data(:,265);

LKneeRot = data(:,267);

RKneeFlex = data(:,279);

RKneeAbAd = data(:,278);

RKneeRot = data(:,280);

LAnkleFlex = data(:,259);

LankleProSup = data(:,260);

RAnkleFlex = data(:,272);

RAnkleProSup = data(:,273);

LFootProg = data(:,45);

RFootProg = data(:,256);

MomentLHipAbAd = data(:,201);

MomentLHipFlex = data(:,202);

MomentLHipRot = data(:,203);

MomentRHipAbAd = data(:,214);

MomentRHipFlex = data(:,215);

MomentRHipRot = data(:,216);

MomentLKneeAbAd = data(:,204);

MomentLKneeFlex = data(:,205);

MomentLKneeRot = data(:,206);

MomentRKneeAbAd = data(:,217);

MomentRKneeFlex = data(:,218);

MomentRKneeRot = data(:,219);

MomentLAnkleFlex = data(:,198);

MomentRAnkleFlex = data(:,211);

AVE_Peak_LHipFlexMoment = mean(pksMntLHipFlex);

SDLHipFlexMoment = std(pksMntLHipFlex);

MomentLHipFlexInv = MomentLHipFlex*-1;

[pksMntLHipFlexInv locsMntLHipFlexInv] = findpeaks(MomentLHipFlexInv,'minpeakdistance',30,'minpeakheight',0.1);

AVE_Peak_LHipFlexMomentInv = mean(pksMntLHipFlexInv);

SDLHipFlexMomentInv = std(pksMntLHipFlexInv);

[pksMntRHipFlex locsMntRHipFlex] = findpeaks(MomentRHipFlex,'minpeakdistance',30,'minpeakheight',0.1);

AVE_Peak_RHipFlexMoment = mean(pksMntRHipFlex);

SDRHipFlexMoment = std(pksMntRHipFlex);

MomentRHipFlexInv = MomentRHipFlex*-1;

[pksMntRHipFlexInv locsMntRHipFlexInv] = findpeaks(MomentRHipFlexInv,'minpeakdistance',30,'minpeakheight',0.1);

AVE_Peak_RHipFlexMomentInv = mean(pksMntRHipFlexInv);

SDRHipFlexMomentInv = std(pksMntRHipFlexInv);

[pksMntLHipAbAd locsMntLHipAbAd] = findpeaks(MomentLHipAbAd,'minpeakdistance',30,'minpeakheight',0.1);

AVE_Peak_LHipAbAdMoment = mean(pksMntLHipAbAd);

SDLHipAbAdMoment = std(pksMntLHipAbAd);

MomentLHipAbAdInv = MomentLHipAbAd*-1;

[pksMntLHipAbAdInv locsMntLHipAbAdInv] = findpeaks(MomentLHipAbAdInv,'minpeakdistance',30,'minpeakheight',0.1);

AVE_Peak_LHipAbAdMomentInv = mean(pksMntLHipAbAdInv);

SDLHipAbAdMomentInv = std(pksMntLHipAbAdInv);

[pksMntRHipAbAd locsMntRHipAbAd] = findpeaks(MomentRHipAbAd,'minpeakdistance',30,'minpeakheight',0.1);

AVE_Peak_RHipAbAdMoment = mean(pksMntRHipAbAd);

SDRHipAbAdMoment = std(pksMntRHipAbAd);

MomentRHipAbAdInv = MomentRHipAbAd*-1;

[pksMntRHipAbAdInv locsMntRHipAbAdInv] = findpeaks(MomentRHipAbAdInv,'minpeakdistance',30,'minpeakheight',0.1);

AVE_Peak_RHipAbAdMomentInv = mean(pksMntRHipAbAdInv);

SDRHipAbAdMomentInv = std(pksMntRHipAbAdInv);

[pksMntLHipRot locsMntLHipRot] = findpeaks(MomentLHipRot,'minpeakdistance',30,'minpeakheight',0.1);

AVE_Peak_LHipRotMoment = mean(pksMntLHipRot);

SDLHipRotMoment = std(pksMntLHipRot);

MomentLHipRotInv = MomentLHipRot*-1;

[pksMntLHipRotInv locsMntLHipRotInv] = findpeaks(MomentLHipRotInv,'minpeakdistance',30,'minpeakheight',0.1);

AVE_Peak_LHipRotMomentInv = mean(pksMntLHipRotInv);

SDLHipRotMomentInv = std(pksMntLHipRotInv);

[pksMntRHipRot locsMntRHipRot] = findpeaks(MomentRHipRot,'minpeakdistance',30,'minpeakheight',0.1);

AVE_Peak_RHipRotMoment = mean(pksMntRHipRot);

SDRHipRotMoment = std(pksMntRHipRot);

MomentRHipRotInv = MomentRHipRot*-1;

[pksMntRHipRotInv locsMntRHipRotInv] = findpeaks(MomentRHipRotInv,'minpeakdistance',30,'minpeakheight',0.1);

AVE_Peak_RHipRotMomentInv = mean(pksMntRHipRotInv);

SDRHipRotMomentInv = std(pksMntRHipRotInv);

[pksMntLKneeFlex locsMntLKneeFlex] = findpeaks(MomentLKneeFlex,'minpeakdistance',30,'minpeakheight',0.1);

AVE_Peak_LKneeFlexMoment = mean(pksMntLKneeFlex);

SDLKneeFlexMoment = std(pksMntLKneeFlex);

MomentLKneeFlexInv = MomentLKneeFlex*-1;

[pksMntLKneeFlexInv locsMntLKneeFlexInv] = findpeaks(MomentLKneeFlexInv,'minpeakdistance',30,'minpeakheight',0.1);

AVE_Peak_LKneeFlexMomentInv = mean(pksMntLKneeFlexInv);

SDLKneeFlexMomentInv = std(pksMntLKneeFlexInv);

[pksMntRKneeFlex locsMntRKneeFlex] = findpeaks(MomentRKneeFlex,'minpeakdistance',30,'minpeakheight',0.1);

AVE_Peak_RKneeFlexMoment = mean(pksMntRKneeFlex);

SDRKneeFlexMoment = std(pksMntRKneeFlex);

MomentRKneeFlexInv = MomentRKneeFlex*-1;

[pksMntRKneeFlexInv locsMntRKneeFlexInv] = findpeaks(MomentRKneeFlexInv,'minpeakdistance',30,'minpeakheight',0.1);

AVE_Peak_RKneeFlexMomentInv = mean(pksMntRKneeFlexInv);

SDRKneeFlexMomentInv = std(pksMntRKneeFlexInv);

[pksMntLKneeAbAd locsMntLKneeAbAd] = findpeaks(MomentLKneeAbAd,'minpeakdistance',20,'minpeakheight',0.1);

AVE_Peak_LKneeAbAdMoment = mean(pksMntLKneeAbAd);

SDLKneeAbAdMoment = std(pksMntLKneeAbAd);

MomentLKneeAbAdInv = MomentLKneeAbAd*-1;

[pksMntLKneeAbAdInv locsMntLKneeAbAdInv] = findpeaks(MomentLKneeAbAdInv,'minpeakdistance',20,'minpeakheight',0.01);

AVE_Peak_LKneeAbAdMomentInv = mean(pksMntLKneeAbAdInv);

SDLKneeAbAdMomentInv = std(pksMntLKneeAbAdInv);

these

[pksMntRKneeAbAd locsMntRKneeAbAd] = findpeaks(MomentRKneeAbAd,'minpeakdistance',20,'minpeakheight',0.1);

AVE_Peak_RKneeAbAdMoment = mean(pksMntRKneeAbAd);

SDRKneeAbAdMoment = std(pksMntRKneeAbAd);

MomentRKneeAbAdInv = MomentRKneeAbAd*-1;

[pksMntRKneeAbAdInv locsMntRKneeAbAdInv] = findpeaks(MomentRKneeAbAdInv,'minpeakdistance',20,'minpeakheight',0.1);

AVE_Peak_RKneeAbAdMomentInv = mean(pksMntRKneeAbAdInv);

SDRKneeAbAdMomentInv = std(pksMntRKneeAbAdInv);

[pksMntLKneeRot locsMntLKneeRot] = findpeaks(MomentLKneeRot,'minpeakdistance',30,'minpeakheight',0.01);

AVE_Peak_LKneeRotMoment = mean(pksMntLKneeRot);

SDLKneeRotMoment = std(pksMntLKneeRot);

MomentLKneeRotInv = MomentLKneeRot*-1;

[pksMntLKneeRotInv locsMntLKneeRotInv] = findpeaks(MomentLKneeRotInv,'minpeakdistance',30,'minpeakheight',0.01);

AVE_Peak_LKneeRotMomentInv = mean(pksMntLKneeRotInv);

SDLKneeRotMomentInv = std(pksMntLKneeRotInv);

[pksMntRKneeRot locsMntRKneeRot] = findpeaks(MomentRKneeRot,'minpeakdistance',30,'minpeakheight',0.01);

AVE_Peak_RKneeRotMoment = mean(pksMntRKneeRot);

SDRKneeRotMoment = std(pksMntRKneeRot);

MomentRKneeRotInv = MomentRKneeRot*-1;

[pksMntRKneeRotInv locsMntRKneeRotInv] = findpeaks(MomentRKneeRotInv,'minpeakdistance',30,'minpeakheight',0.01);

AVE_Peak_RKneeRotMomentInv = mean(pksMntRKneeRotInv);

SDRKneeRotMomentInv = std(pksMntRKneeRotInv);

[pksMntRAnkleFlex locsMntRAnkleFlex] = findpeaks(MomentRAnkleFlex,'minpeakdistance',30,'minpeakheight',0.1);

AVE_Peak_RAnkleFlexMoment = mean(pksMntRAnkleFlex);

SDRAnkleFlexMoment = std(pksMntRAnkleFlex);

MomentRAnkleFlexInv = MomentRAnkleFlex*-1;

[pksMntRAnkleFlexInv locsMntRAnkleFlexInv] = findpeaks(MomentRAnkleFlexInv,'minpeakdistance',30,'minpeakheight',0.1);

AVE_Peak_RAnkleFlexMomentInv = mean(pksMntRAnkleFlexInv);

SDRAnkleFlexMomentInv = std(pksMntRAnkleFlexInv);

[pksMntLAnkleFlex locsMntLAnkleFlex] = findpeaks(MomentLAnkleFlex,'minpeakdistance',30,'minpeakheight',0.1);

AVE_Peak_LAnkleFlexMoment = mean(pksMntLAnkleFlex);

SDLAnkleFlexMoment = std(pksMntLAnkleFlex);

MomentLAnkleFlexInv = MomentLAnkleFlex*-1;

[pksMntLAnkleFlexInv locsMntLAnkleFlexInv] = findpeaks(MomentLAnkleFlexInv,'minpeakdistance',30,'minpeakheight',0.1);

AVE_Peak_LAnkleFlexMomentInv = mean(pksMntLAnkleFlexInv);

SDLAnkleFlexMomentInv = std(pksMntLAnkleFlexInv);
